# Discussion

**Published:** 1918-12

**Authors:** 


**Discussion.**

MAJOR GENERAL SIR DAVID BRUCE said that at the beginning of the war orders with regard to the prophylactic injection of anti-tetanic serum were frequent in connection with wounds, but it was not recognized that trench foot was a wound. One day it was found that, within a fortnight, 15 cases of tetanus originated in cases of trench foot in which no one thought that prophylactic antitoxin was necessary. The 15 cases made the surgeons open their eyes.

The speaker wrote a paper to the British Medical Journal calling attention to this fact, and as there was no time to be lost he took the first opportunity when the reports on only 8 of these 15 cases had come in. Of these 8 cases 6 died, and the duration of the disease in these 6 cases was only two and a half days, showing that if the prophylactic injection of anti-tetanus is omitted there is a return of the old tetanus of pre-serum days, which kills men in from 2 to 3 days. Therefore in all cases of trench foot, see that the man has at once a thorough dose of antitoxin.

LIEUTENANT-COLONEL NORRIS described his experience at Base Hospital N° 10, where he undertook studies of trench foot.

His blood pressure observations showed invariably that in cases of trench foot the blood pressure is higher in the legs than in the arms, and it seems quite definitely established that trench foot is essentially a vaso-motor condition.

LIEUTENANT-COLONEL NORRIS decided to try treating these cases with potassium iodide, and he was surprised at the results obtained. The administration of potassium iodide relieves pain in a most remarkable manner, and this treatment is distinctly beneficial. The men who received the potassium iodide went back to duty sooner than those who were not put on this line of treatment.

Lieutenant-Colonel J. Goldthwait said that the experiences which he and his colleagues had last winter made everyone feel that the essential element in the treatment of trench foot is that which had been mentioned so clearly, that the feet should be kept dry, and so treated that the moisture, which of course is increased when one stands for a long time, can get away from the feet. For that reason it was felt that whale-oil and other oils of that sort were undesirable because they made a coating and prevented the moisture from getting away from the skin.

Oil should only be used when the men stand in water and when gum boots are not available, or when it is impossible to remove the gum boots for a long time. If gum boots are not possible and the men must stand in water, oil put over the feet keeps some of the moisture from getting in.

With well-oiled boots there is rarely any use for whale-oil on the skin. Washing, followed by powdering and the issuing of warm socks, is the best prevention of trench foot. It seems important to have some medical officers go around among the different units and stimulate the interest of the commanding officers so that proper provision may be made for bathing and powdering the men's feet.

Major Cannon attributed changes in circulation as largely and almost wholly due to vaso-motor disturbances. Anything that will interrupt the flow of blood through the capillaries will alter the capillary wall and will lead to greater permeability and consequent edema and stagnation. Other points than arterial circulation should be kept in mind.

Colonel Cummins said that in studying existing Regulations on the subject of precautions against trench foot, he had noticed the constant recurrence of the words " if possible "; the truth being that nearly all the precautions that were practically essential were only occasionally possible under the conditions prevailing in trench warfare.

As long as men were obliged to remain for protracted periods in wet trenches, this disability would continue to be common. The more important prophylactic measures were not in the hands of the Medical Service, but were dominated by the conditions of battle at any given time. These were :

*a*) The provision of dry standing in the trenches, or

*b*) The cutting down of the time spent by the troops in wet trenches.

It was sometimes impossible to ensure that the men reached the trenches dry and not overtired. As an instance of actual conditions which led to a high incidence of trench foot, Colonel Cummins quoted the case of a British division arriving from India, and being sent direct for duty in France early in 1915. The distance from the rest billets to the front trenches was considerable, and the men had to march 17 miles along bad roads and through wet communication trenches before reaching the trenches in which they were to serve. They arrived thoroughly tired and covered with perspiration, in addition to having their legs wet. Reliefs only took place at long intervals. Under these circumstances the high incidence of trench foot, which actually occurred, was inevitable.

Referring to the " long gum boots ", Colonel Cummins pointed out that while these were very useful for men who could put them on dry, and were not obliged to walk long distances in them, they were apt to become filled with perspiration if the men exerted themselves while wearing them. One division, shortly after the issue of these boots in 1916, reported that they had found the " real cause of trench foot " ; they considered it to be the long gum boots.

The essential in preventing this condition is to preserve a layer of air between the skin and the covering of the feet and legs. This layer of air was lost when men stood in wet trenches in ordinary boots; but it was equally lost when the socks became soaked with perspiration inside the gum boots.

